# Supplementary material for: Multi-Omics and Experimental Validation Identify GPX7 and Glutathione-Associated Oxidative Stress as Potential Biomarkers in Ischemic Stroke
Source: Antioxidants (Basel). 2025 May 30;14(6):665. doi: 10.3390/antiox14060665 (PMC12189666; doi:10.3390/antiox14060665)
Supplement: Supplementary file 1 [file antioxidants-14-00665-s001.zip › antioxidants-3640153-supplementary.pdf]

Table S1. Primer sequences used in this study.

Genes		Primers (5′–3′)
Gpx7	F	TCCGAGCAGGACTTCTACGAC
	R	TCTCCCTGTTGGTGTCTGGTT
Il6	F	TAGTCCTTCCTACCCCAATTTCC
	R	TTGGTCCTTAGCCACTCCTTC
Il1-beta	F	GCAACTGTTCCTGAACTCAACT
	R	ATCTTTTGGGGTCCGTCAACT
GAPDH	F	GAGTCAACGGATTTGGTCGT
	R	GACAAGCTTCCCGTTCTCAG
